# Patient motivations surrounding participation in phase I and phase II clinical trials of cancer chemotherapy

**DOI:** 10.1038/sj.bjc.6602423

**Published:** 2005-03-15

**Authors:** Z A Nurgat, W Craig, N C Campbell, J D Bissett, J Cassidy, M C Nicolson

**Affiliations:** 1 Pharmacy Department, Aberdeen Royal Infirmary, Foresterhill Site, Aberdeen AB25 2ZN, UK; 2 Department of General Surgery, Aberdeen Royal Infirmary, Foresterhill Site, Aberdeen AB25 2ZN, UK; 3 Department of General Practice and Primary Care, Foresterhill Health Centre, Aberdeen University Medical School, University of Aberdeen, Westburn Road, Foresterhill, Aberdeen AB25 2AY, UK; 4 ANCHOR Unit, Department of Clinical Oncology, Aberdeen Royal Infirmary, Foresterhill Site, Aberdeen AB25 2ZN, UK; 5 Beatson Oncology Center, The Western Infirmary, Dunbarton Road, Glasgow G11 6NT, UK; 6 ANCHOR Unit, Department of Medical Oncology, Aberdeen Royal Infirmary, Foresterhill Site, Aberdeen AB25 2ZN, UK

**Keywords:** informed consent, phase I/II cancer clinical trials, patient motivations

## Abstract

Successful advances in the treatment of advanced malignant diseases rely on recruitment of patients into clinical trials of novel agents. However, there is a genuine concern for the welfare of individual patients. The aim of this study was to examine motives of patients entering early clinical trials of novel cancer therapies. Questionnaire survey with both open- and close-ended questions. The patients were surveyed after they had given informed consent and before or during the first cycle of treatment. In all, 38 phase I/II trial patients participated and completed the survey. Obtaining possible health benefit was listed by 89% as being a ‘very important’ factor in their decision to participate, with only 17% giving reasons of helping future cancer patients and treatment. Other items cited as a ‘very important’ motivating factor were ‘trust in the doctor’ (66%), ‘being treated by the latest treatment available’ (66%), ‘better standard of care and closer follow-up’ (61%), and ‘closer monitoring of patients in trials’ (58%). Only 47% patients indicated that someone had explained to them about any ‘reasonable’ alternatives to the trial. In total, 71% strongly agreed that ‘surviving for as long time as possible was the most important thing (for them)’. Nearly all (97%) indicated that they knew the purpose of the trial and had enough time to consider participation in the trial (100%). In this survey, most patients entering phase I and II clinical trials felt they understood the purpose of the research and had given truly informed consent. Despite this, most patients participated in the hope of therapeutic benefit, although this is known to be a rare outcome in this patient subset. Trialists should be aware, and take account of the expectations that participants place in trial drugs.

Successful advances in the treatment of advanced malignant diseases are reliant on recruitment of patients into clinical trials of novel agents (Tannock, 1995). These studies are normally restricted to patients with advanced malignant disease that is refractory to standard therapy. There is, however, genuine concern with regard to the welfare of the individual patients in such clinical trials (The National Commission, 1979; Faden *et al*, 1986).

Although all phases of clinical trials are associated with ethical issues, there are particular problems with phase I and phase II trials. Firstly, preclinical experiments provide only limited information from which to predict the dose, schedule, toxicity, and anticancer activity of the drug in man. Patients in phase I trials, therefore, run the risk of receiving doses of drug that have no biological effect or, alternatively, excessively high doses with the risk of serious toxicity. Although phase I trials of newer agents have the potential to minimise the risk of toxicity by using more rational biological end points, most current trials still use the traditional end points of toxicity for selection of the recommended phase II dose (Parulekar and Eisenhauer, 2004). Similar risk can also occur in phase II trials. Secondly, this is a particularly vulnerable group of patients, who are usually well aware of their advanced malignant disease, short life expectancy, and lack of established treatment options. Ethically, treatment of these patients should ensure that they are well informed about potential risks and benefits associated with trial participation as well as the alternatives to trial participation.

Patients' understanding of the difference between therapeutic and nontherapeutic research has been called into question (Appelbaum *et al*, 1987; Lidz *et al*, 2004). The published literature on patient motivation to participate in clinical trials suggests that altruism may not be the sole motivating factor; self-interest is also important (Penman *et al*, 1984; Rodenhuis *et al*, 1984; Kodish *et al*, 1992; Daugherty *et al*, 1995; Itoh *et al*, 1997; Yoder *et al*, 1997). A recent systematic review, however, has questioned whether participation in clinical trials is of any benefit to participants (Peppercorn *et al*, 2004). Various studies have reported that the chance of therapeutic response for those volunteering to take part in phase I trial is less than 5% (Estey *et al*, 1986; Decoster *et al*, 1990; Von Hoff and Turner, 1991; Smith *et al*, 1996; Roberts *et al*, 2004). In phase II clinical trials, the overall objective response rate (partial and complete) is also usually low. Higher response rates (>20–30%) must be observed in breast or small cell lung cancer to make a new drug interesting for further development, as there are numerous drugs already active in these tumour types. In contrast, in tumour types such as glioma and melanoma where there are few effective treatments response rates as low as 5% may still render the drug interesting and potentially useful as a future treatment option.

In this study, we investigated the motivations and inhibiting factors for patients participating in phase I and phase II cancer clinical trials, their understanding of the purpose of the research and alternatives to trial participation, and influences on the decision to enter the trial.

## MATERIALS AND METHODS

Between January 2001 and January 2002, patients with advanced or metastatic cancer attending the Aberdeen and North Centre for Haematology, Oncology and Radiotherapy (ANCHOR) Unit who had given informed consent to participate in clinical trials were invited to take part in a questionnaire study. The local ethical committee approved the study design and the final format of the questionnaire. Patients were identified through the hospital's Medicines Assessment Research Unit (MARU) and the oncology research nurses. The patients were approached as in-patients or outpatients, depending on the nature of the treatment they were receiving.

The survey's principal investigator (PI) asked patients if they would agree to participate in a questionnaire survey that would take around 15–20 min to complete (see Supplementary information). Once verbal approval was obtained, the PI issued the questionnaire before any treatment began. Assurance was given to patients that it would not affect their treatment should they choose not to complete the questionnaire, and that their anonymity would be maintained. The questionnaire was completed in the absence of the research nurses. The questionnaires were returned by mail (stamped addressed envelope was provided where necessary) or hand delivered to the ward or the clinic. A maximum of 1 month was allowed for the return of the questionnaire.

### Study instruments

The questionnaire was adopted from Daugherty *et al* (1995) with the author's permission, but included a new section on the ‘trade-off’ between quality of life (QOL) and long-term survival. Prior to commencing this study, the questionnaire was piloted with 20 patients at a similar stage of recruitment to cancer trials to ensure clarity of meaning.

Analysis of the data was carried out using SPSS for Windows (version 10.0.7 program). The primary statistical analysis was intended to be descriptive in nature. Secondary analysis was performed using nonparametric tests for independent samples with the Mann–Whitney test for two-sided independent samples at the 5% significance level. Accuracy of the data was checked in 100% of the cases by the second author after going through each questionnaire and checking all the completed data input.

## RESULTS

### Patient accrual

In all, 104 patients were approached for the study. Of these, 63 patients were participants of phase III trials and were ineligible for the study. Of the remaining 41 patients, 14 were participants in phase II trials and 27 in phase I trials. We found that we had double counted two patients who had completed the questionnaire twice (for different trials), so we excluded their second questionnaires, and another that was largely incomplete, leaving a final cohort of 38 participants. Tables 1 and 2 show the demographic and general health characteristics of the participants.

### Questionnaire responses

Overall 98% of the close-ended questions were answered. In all, 92% had answered the open-ended question ‘Can you tell us the main reason that you are participating in this clinical trial?’, and these responses were analysed for key words or phrases (hope of remission, help me/help others, improve health, reduce tumour) to look for clues for patient motivation. Only six (17%) gave altruistic reasons of helping future cancer patients and treatment (‘If it can help other cancer patients, then that's good’), whereas 22 (58%) gave answers indicating some hope of therapeutic response (‘To help me get better’, ‘Had two different chemotherapies before but didn't have the desired effect’).

In the closed questions, 30 (82%) listed helping future cancer patients as being a ‘very important’ motivating factor for participating in the trial. Other important motivating items cited were ‘possible health benefit’ (89%), ‘trust in the doctor’ (66%), ‘trust in nurses’ (76%), ‘being treated by the latest treatment available’ (66%), ‘better standard of care and closer follow-up’ (61%), ‘being likely to obtain more information about my condition’ (58%), and ‘closer monitoring of patients in trials’ (58%).

### Patient expectations

In total, 35 (92%) thought patients benefit from clinical trials and 33 (87%) rated highly the possibility of personal clinical benefit. If offered the chance to participate in a future trial, 18 (47%) indicated ‘probably yes’ and 16 (42%) ‘definitely yes’. Chemotherapy-naïve patients were more likely to have positive expectations of benefit from participating in the trial than those patients who had previously had chemotherapy (100 *vs* 78%) (*z*=1.91; *P*=0.05). Men were significantly more likely than women (100 *vs* 64%) to have positive expectations from participating in the trials (*z*=3.09; *P*<0.01).

### QOL *vs* long-term survival

In the questions about QOL and length of survival, 27 (71%) strongly agreed that ‘surviving for as long time as possible is the most important thing’ (for them). In all, 21 (55%) strongly disagreed that maintaining QOL was less important. A total of 23 (60%) strongly agreed that they ‘would rather maintain a better QOL for a shorter term than suffer somewhat for longer’, with nine (24%) neither agreeing nor disagreeing (Table 3).

### Sources of information

Table 4 shows the sources of information that the patients had used since diagnosis. In all, 29 (76%) sought more information about their illness and treatment options after their diagnosis, 17 (45%) prior to the treatment and 19 (50%) during treatment. A total of 12 (32%) obtained more information when looking for a different treatment. In total, 20 (53%) had contacted relatives, friends, and other people for more information, while the Internet and the MacMillan or Marie Curie organisations were used by 26%. In all, 30 (82%) had discussed their prognosis with their oncologist and 33 (87%) said they understood their prognosis.

### Comprehension

When asked through close-ended questions, 37(97%) indicated that they gave informed consent, with 36 (95%) having understood all or most of the trial information given to them (Table 5). Virtually all patients said that someone had explained that the trial was part of medical research, and told them the type of treatment they would be getting. Patients felt they had had plenty of time to think things over. Nearly all patients (97%) said that the side effects they might experience and the risks involved (89%) were explained to them. A total of 18 (47%) patients indicated that someone had explained to them about any ‘reasonable’ alternatives.

Phase I trial patients were significantly more likely than patients on phase II trials (65 *vs* 25%) to indicate that no ‘reasonable’ alternatives to having this treatment were explained (*z*=2.287; *P*<0.05). Patients who previously had chemotherapy were significantly more likely than chemotherapy-naïve patients (70 *vs* 27%) to indicate that no reasonable alternatives to treatment were explained to them (*z*=2.554; *P*<0.01).

### Influences

Most (97%) when asked through close-ended questions made up their own mind to participate in the trial, although doctors at the cancer centre, family, and family doctors (to a lesser extent) were also influential (Table 6). In all, 20 (52%) found the decision to participate in the trial ‘easy’. In total, 31 (82%) made the decision to participate completely on their own and seven (18%) partially.

## DISCUSSION

We found that most participants felt they were well informed and that their decisions about trial participation were made independently. The main area of concern was that only half of the patients felt that they had ‘reasonable’ alternatives to trial participation explained. Those not aware of ‘reasonable’ alternative treatment were, however, predominantly participants in phase I trials, where the only alternative was likely to have been an alternative experimental therapy or best supportive care.

Our study has some limitations – in particular, it is a small study from a single institution – but the participants are likely to be broadly representative of those in phase I and II clinical trials. On direct questioning, the two main motivational factors for trial participation were possible health benefit and helping future cancer patients. The differences we found in responses to open- and close-ended questions suggest, however, that participants' main motivation was personal health gain. Whereas phrasing of a closed question might influence the response, open-ended question may more freely elicit patients' true feelings. For example, Cox (2003) found that QOL of patients in phase I and phase II cancer drug trials as measured by closed question instruments was unaffected by trial participation, whereas qualitative data from in-depth interviews demonstrated considerable physical and psychological impact from experimental chemotherapy. Our finding that personal health benefit was the most important motivation and expectation for most patients is in line with previous findings (Daugherty *et al*, 1995; Yoder *et al*, 1997; Cheng *et al*, 2000). How then do we explain this apparent mismatch in a group of patients who report being well informed?

Patients may have interpreted the information they received optimistically. Cox (2002) reported that patients interpreted the wording of information sheets optimistically, for example, the words ‘study’, ‘new’, or ‘American’ treatment were taken to mean ‘better’ than conventional treatments. The positive presentation of trials was reinforced by faith patients place in the decision of their consultants to offer this treatment. Our results with regard to trust in doctors are similar to those obtained by Penman *et al* (1984) and Daugherty *et al* (1995). This vulnerable group of patients may be at risk of pressure by their doctors to enter studies, but we found few who did not decide independently to participate. Albrecht *et al* (1999) found that patients were more likely to consent to the trial when their physician verbally presented items normally included in an informed consent document and when they behaved in a ‘reflective, patient-centred, supportive, and responsive manner’.

Cheng *et al* (2000) found that participants in phase I clinical have high expectations regarding the success of experimental therapy and discount potential toxicity, and that patients perceive potential benefits and toxicities differently than health-care professionals. We observed similar findings in that most patients rated highly the possibility of personal benefit. Slevin *et al* (1990) found that patients with cancer are willing to accept treatment with cytotoxics for lower chances of benefit than those thought acceptable by their physicians or nurses.

Many patients maintain hope and optimism despite advanced cancer. In a qualitative study, we found that many patients needed to maintain some degree of hope (Bain *et al*, 2002) and others have reported the same (Leydon *et al*, 2000). For a patient whose illness has progressed on standard therapy and for whom no other established therapy is available, a less than 5% chance of therapeutic benefit could be regarded as reasonable justification for study entry. Furthermore, with increased use of targeted therapy, the risk of toxic effects experienced by participants in phase I trials is improving (Roberts *et al*, 2004).

Patients are attracted to a clinical trial by ‘being treated by a doctor with a specialist interest in the disease and encouraged by the possibility that their progress will be monitored closely’ (Slevin *et al*, 1995). High-quality care and closer monitoring are realistic expectations for phase I and phase II trials. Patients in these trials have a dedicated team of nurses, pharmacists, and doctors looking after them and have close contact with the oncology department. This provides continuity of care, prompt attention to symptom control, and a high level of support.

Our findings confirm that when patients believe they are well informed, their understanding of potential risks (predominantly to QOL) and benefits (mostly in terms of the small potential for benefit to survival) are key to their decisions to participate in phase I and phase II clinical trials. In an attempt to illuminate this further, we asked questions on the trade offs they would make between survival and QOL. Their responses demonstrate the importance they placed on both survival and QOL, but their trade offs were inconsistent with each other (most strongly agreed that survival for as long as possible was most important, and also that QOL was more important than survival). Instead, their responses appear to be in line with the way statements were framed, suggesting that patients' consideration of risks and benefits, even when fully understood, is vulnerable to manipulation (Thornton, 2003).

Overall, we have found that nearly all patients recruited to phase I and II clinical trials felt they were fully informed about the research and, with the possible exception of alternative treatments, appeared to be well informed on direct questioning. Despite this, most took part primarily in the hope of therapeutic benefit. Trialist clinicians should take account of patients' potential misconceptions about early clinical trials of anticancer agents during recruitment, and should ensure clarity and an honest approach to such vulnerable patients.

## Figures and Tables

**Table 1:** Demographics of the patients (*N*=38)

**Characteristic**	***N* (%)**
*Gender*	
Male	24 (63)
Female	14 (37)
	
*Age (year)*	
37–49	7 (18)
50–59	12 (32)
60–69	13 (34)
70–79	6 (16)
	
*Marital status*	
Single	6 (16)
Married	24 (63)
Separated/divorced/widowed	8 (21)
	
*Education*	
High school	21 (55)
College	8 (21)
University/postgraduate	3 (8)
Unknown	6(16)
	
*Employment*	
Full/part-time employment	11 (29)
Unemployed	2 (5)
Disabled	3 (8)
Housewife	4 (10)
Retired	16 (42)
Unknown	2 (5)
	
*Tumour type*	
Colorectal	15 (39)
Oesophageal	6 (16)
Lung (NSCLC)	4 (10)
Mesothelioma	4 (10)
Gastric	2 (5)
Leiomyosarcoma	2 (5)
Unknown primary	2 (5)
Other	3 (8)
	
*Previous chemotherapy*	
Yes	23 (60)
No	15 (39)
	
*Previous trial participation*	
Yes	7 (18)
No	31 (82)

**Table 2:** Patient health status (*N*=38)

	**Never (%)**	**Rarely (%)**	**Sometimes (%)**	**Frequently (%)**	**Always (%)**
Needing help to travel about	55	13	16	3	13
Staying indoors all day due to health	53	8	24	10	5
Being in bed or chair most day	50	10	32	5	3
Not being able to do vigorous activities	32	11	5	16	34
Having trouble climbing/walking	42	11	26	18	3
Having trouble bending/lifting	42	16	24	8	10
Needing help with eating/bath	89	3	5	3	
Having trouble working	34	18	32	10	5

**Table 3:** QOL *vs* long-term survival (*N*=38)

	**Strongly AGREE (%)**	**Agree somewhat (%)**	**Neither Agree nor Disagree (%)**	**Disagree Somewhat**	**Strongly DISAGREE (%)**
Surviving for as long a time as possible is the MOST IMPORTANT thing for me	71	11	11	3	3
Maintaining QOL is LESS IMPORTANT for me	8	8	10	10	55
I would rather maintain a better quality of life for a shorter time than suffer somewhat for longer	60	11	24	5	0

QOL=quality of life.

In the grid above, there are three statements: please indicate with a tick in the appropriate column the extent to which you agree/disagree with each of the three statements.

**Table 4:** Sources of information (*N*=38)

**Question**	**Yes**	**(%)**
*I got more information about my illness/treatment options.*		
Just after diagnosis	29	(76)
Before treatment	17	(45)
During treatment	19	(50)
When looking for a different treatment	12	(32)
		
*I contacted.*		
The National Cancer Alliance	—	—
Literature (books/journals, other than hospital ones)	9	(24)
The Internet	10	(26)
Relatives, friends, other people	20	(53)
Patient support groups, for example, CLAN, BACUP	7	(18)
MacMillan or Marie Curie organisations	10	(26)
Other organisations, for example, Cancer Research Campaign	6	(16)
The hospital, outside my appointment times	5	(13)
		
Were you satisfied with the amount of information received?	29	(76)
Has a doctor ever spoken with you about your prognosis?	31	(82)
Do you think that you understand what your prognosis is?	33	(87)

**Table 5:** Comprehension of the purpose of phase I or phase II clinical trials (*N*=38)

**Purpose**	**Yes**	**(%)**
*Did someone explain.*		
The type of treatment you would get	38	(100)
The purpose of this treatment	37	(97)
Unintended side effects you may experience	37	(97)
Risks involved in having this treatment	34	(89)
Benefits you may experience in having this treatment	34	(89)
That this trial was part of medical research	38	
Any reasonable alternatives to having this treatment	18	(47)
Do you think you were well informed?	37	(97)
Were you able to ask enough questions?	36	(95)
		
Did you understand all of the trial information	18	(47)
Did you understand most of the trial information	18	(47)
Did you understand some of the trial information	2	(6)
Did you understand almost none of the trial information	0	—
		
*Additionally did you understand.*		
How the research trial would achieve this	34	(89)
How the trial worked	33	(87)
How the trial could help patients now	34	(89)
How the trial could help future cancer patients	33	(87)
The possible benefits and risks to patients in the trial	34	(89)
That you are free to withdraw from the trial at any point in time	37	(97)
That the trial is ethically approved and regulated	34	(89)
		
Did you give informed consent	37	(97)
Did you have enough time to consider	38	(100)
Did you know the purpose of this trial	37	(97)

**Table 6:** Patients' decision-making and influences for participation in the trial (*N*=38)

	**Yes (%)**
Did you make up your own mind to participate in this trial?	37 (97)
	
*With whom did you discuss your decision.*	
Doctors at the cancer centre	31 (82)
Nurses	6 (16)
Family doctor	14 (37)
Family	27 (71)
Friends	9 (24)
	
*Was the decision.*	
Hard	6 (16)
Easy	20 (52)
Or somewhat in between?	12 (32)
	
*Would you say you made your own decision.*	
Completely	31 (82)
Partially	7 (18)
Or almost not all?	—
