# Supplementary material for: Patient motivations surrounding participation in phase I and phase II clinical trials of cancer chemotherapy
Source: Br J Cancer. 2005 Mar 15;92(6):1001–5. doi: 10.1038/sj.bjc.6602423 (PMC2361930; doi:10.1038/sj.bjc.6602423)
Supplement: Supplementary Information [file 92-6602423x1.doc]

# **Appendix 1**

# CODE__ __ __

# A Study of Patient Motivations and Health Beliefs Surrounding Participation in Clinical Trials of Cancer Chemotherapy

# **Questionnaire**

# For patients immediately post-enrolment in a clinical trial of cancer chemotherapy

#

We are very grateful for your participation in this questionnaire, and are interested in finding out your attitudes toward health care and clinical trials. There are no right or wrong answers; all we are looking for are opinions.

###### Section 1: Health Status

We’d like to know about health problems you might have other than the cancer that we are treating you for. Do you have any of the following illnesses at the present time, and if so, how much does it interfere with your activities? Please tick the columns that best reflect your answer.

|  |  |  |  | NO | YES - and it interferes | |  |  | |  |  |
| --- | --- | --- | --- | --- | --- | --- | --- | --- | --- | --- | --- |
|  |  |  |  |  |  | Not at all | Somewhat | A great deal | | |
| Other Cancers | |  |  |  |  |  |  |  | |  |
| Arthritis/Other Joint Problems | | |  |  |  |  |  |  | |  |
| Glaucoma | |  |  |  |  |  |  |  | |  |
| Emphysema/Chronic Bronchitis | | | |  |  |  |  |  | |  |
| High Blood Pressure | | |  |  |  |  |  |  | |  |
| Heart Problems | |  |  |  |  |  |  |  | |  |
| Circulatory Problems | | |  |  |  |  |  |  | |  |
| Diabetes |  |  |  |  |  |  |  |  | |  |
| Stomach/Intestinal Problems | | |  |  |  |  |  |  | |  |
| Osteoporosis | |  |  |  |  |  |  |  | |  |
| Liver/Kidney Disease | | |  |  |  |  |  |  | |  |
| Stroke |  |  |  |  |  |  |  |  | |  |
| Depression | |  |  |  |  |  |  |  | |  |

Sometimes medical problems may affect everyday activities. For each of the items listed, please rate how often this has happened to you **in the past two weeks because of your health.**

Needing help from

someone to travel

around the community

Staying indoors all day

because of your health

Being in a bed or chair

most of the day

Not being able to do

vigorous activities

anymore e.g. sports

Having trouble walking

100 yards/climbing

stairs

Having trouble bending

or lifting

Having trouble working

on a job, doing

housework

Needing help with

eating, dressing, bathing

or toileting

Never Rarely Sometimes Frequently Always

### Section 2) Why you are Participating in this Trial

Can you tell us the main reason that you are participating in this clinical trial?

…………………………………………………………………………………………………………………………………………………………………………………………………………................................................................

On the next page there is a table containing a list of factors which you may have considered before agreeing to participate in the drug trial; some may have encouraged you, others discouraged you. Please can you indicate with a tick in the appropriate column how important these were for you. IF A REASON PROVIDED DID NOT APPLY TO YOU, PLEASE LEAVE THE ROW BLANK.

Not Slightly Moderately Very

Important Important Important Important

Wanted to be part of research

Needing to have more tests than

if not on trial therapy

Side effects of treatment

Trust in the doctor

Helping future cancer patients

Doctor may be more concerned

about the trial than about me

Family encouraged me to participate

in the trial

Not being able to choose the treatment

myself

Possible health benefit from therapy

Getting a better standard of care,

closer follow up

Frightened by the forms I had to fill in

A doctor didn’t want me to participate

in the trial

Time/money spent travelling to clinic

Trust in the trial

No better option

A doctor encouraged me to participate

in the trial

Experimental status of research

Wanting a treatment that was only

available on study

Trust in the nurses

My family didn’t want me to participate

in the trial

Being treated by the latest treatment

available

Travelling to clinic more often

Too upset by diagnosis to make a good

decision

Closer monitoring of patients n trial

Likely to obtain more information about

my condition

Other? Please State.....................................

If you were offered the chance, in the future, to participate in another trial, how likely would you be to agree?

Definitely not □

(please tick) Probably not □

Probably yes □

Definitely yes □

Do you think patients benefit from trials? YES/NO (please circle)

Do you think that you will benefit from this trial? YES/NO (please circle)

In the grid below there are three statements: please indicate with a tick in the appropriate column the extent to which you agree/disagree with each of the three statements.

Strongly AGREE

Agree somewhat

Neither Agree nor Disagree

Disagree Somewhat

Strongly DISAGREE

| Surviving for as long a time as possible is the MOST IMPORTANT thing for me |  |  |  |  |  |
| --- | --- | --- | --- | --- | --- |
| Maintaining quality of life is LESS IMPORTANT for me |  |  |  |  |  |
| I would rather maintain a better quality of life for a shorter time than suffer somewhat for longer |  |  |  |  |  |

**Section 3- Informational status**

#### Sources of Information

#### Many people seek out extra information about their disease/treatment options when they are diagnosed with cancer. Please can you tell us more about what you did:

#### Indicate YES/NO to the following questions: YES NO

#### I got more information just after diagnosis □ □

#### before treatment □ □

#### during treatment □ □

#### when looking for a different treatment □ □

YES NO

I contacted: The National Cancer Alliance □ □

Literature (books/journals, other than hospital ones) □ □

The Internet □ □

Relatives, friends, other people □ □

Patient support groups e.g.CLAN, BACUP □ □

Macmillan or Marie Curie organisations □ □

Other Organisations e.g. the Cancer Research Campaign □ □

The hospital, outside my appointment times □ □

What type of information did you need?………………………………………………………………………………………………………………………………………………………………………………………………………………………………………………………………………………………………………………

Were you satisfied with the amount of information you got?

YES/NO

Has a doctor ever spoken with you about your prognosis / what is going to happen to you?

YES/NO

Do you think that you understand what your prognosis is?

YES/NO

#### Your decision to participate

Did you make up your own mind to participate in this trial? YES/NO (please circle)

With whom did you discuss your decision? Doctors at the cancer centre □

Nurses □

Family Doctor □

Family □

Friends □

Other …………………… □ (please tick)

Was the decision hard □

Easy □

or somewhere in between? □ (please tick)

Would you say you made your own decision completely □

Partially □

or almost not at all? □ (please tick)

Unless ‘completely’, who else made the decision?…………………………………

When you were deciding to participate, did someone explain…

(Tick) YES NO

The type of treatment you would get □ □

The purpose of this treatment □ □

Unintended side effects you may experience □ □

Risks involved in having this treatment □ □

Benefits you may experience in having this treatment □ □

That this trial was part of medical research □ □

Any reasonable alternatives to having this treatment □ □

Before you agreed to trial, do you think you were well informed? YES/NO (please circle)

Were you able to ask enough questions? YES/NO (please circle)

You understood all □ (please tick one)

most of □

some of □

almost none of □

the trial information

Additionally, did you understand -

(please tick) YES NO

What the research trial was aiming to find out □ □

And how the research trial would achieve this □ □

How the trial could help patients now □ □

How the trial could help patients in the future □ □

The possible benefits and risks to patients in the trial □ □

That all the information held about you within the trial is confidential □ □

That you are free to withdraw from the trial at any point in time □ □

That the trial is ethically approved and regulated □ □

Who was in the research team for the trial □ □

And did you have a contact number for someone in the team? □ □

Did you give informed consent? YES/NO (please circle)

Did you have enough time to think things over? YES/NO (please circle)

Do you know what the purpose of this trial is? YES/NO (please circle)

**Section 4: About You:**

Name (last, first – please print) ……………………………………………………………

Date of Birth ……/……/……………

Sex: Male □

Female □

Marital Status: Single □

Married □

Separated □

Divorced □

Widowed □

Ethnic Group: White □

Black/African □

Asian □

Other □

Highest Educational Level Attained: High School □

College □

University □

Postgraduate degree □

Employment (current): full/part-time employed □

housewife □

disabled □

unemployed □

student □

retired □

Please specify your current/most recent employment if applicable……………………

…………………………………………………………………………………………

If current employment, how long have you had this job?………………………………

Children – number?…………………………………………

- ages?……………………………………………

Distance from this clinic: Miles?……………………………………………

Time to get here?…………………………………

Mode of transport?………………………………

___________________________________________________________________________

Thank you very much for taking the time to fill in this questionnaire – it is very helpful for us to look at your responses, so that we can understand what patients need when they are involved with research trials. If you have any questions, please do not hesitate to ask the doctor or nurse at your clinic.

For the investigator to complete:

Patient’s Hospital Unit Number……………………………

Diagnosis ……………………………………

Trial Phase ……………………………………

Trial Name/Code ……………………………… (please specify drugs in study)

……………………………………

Oncologist ……………………………………

Any Previous Therapy YES/NO – please specify if yes……

………………………………………

………………………………………

………………………………………

………………………………………

………………………………………

Any Previous Trials – how many? ………………………
